# Supplementary material for: The architecture of EGFR’s basal complexes reveals autoinhibition mechanisms in dimers and oligomers
Source: Nat Commun. 2018 Oct 18;9:4325. doi: 10.1038/s41467-018-06632-0 (PMC6193980; doi:10.1038/s41467-018-06632-0)
Supplement: Supplementary file 1 — Supplementary Information [file 41467_2018_6632_MOESM1_ESM.pdf]

Supplementary Information

The architecture of EGFR's basal complexes reveals autoinhibition mechanisms in dimers and oligomers

Laura C. Zanetti-Domingues[1], Dimitrios Korovesis[1], Sarah R. Needham[1], Christopher J. Tynan[1], Shiori Sagawa[2], Selene K. Roberts[1], Antonija Kuzmanic[3], Elena Ortiz-Zapater[4], Purvi Jain[5], Rob C. Roovers[6], Alireza Lajevardipour[7], Paul M.P. van Bergen en Henegouwen[5], George Santis[4], Andrew H.A. Clayton[7], David T. Clarke[1], Francesco L. Gervasio[3], Yibing Shan[2], David E. Shaw[2,8], Daniel J. Rolfe[1], Peter J. Parker[9,10], and Marisa L. Martin-Fernandez[1]

[1]Central Laser Facility, Research Complex at Harwell, STFC Rutherford Appleton Laboratory, Harwell Oxford, Didcot, Oxford, OX11 0QX UK
[2]D. E. Shaw Research, New York, NY 10036 USA
[3]Department of Chemistry, Faculty of Maths & Physical Sciences, University College London, London, WC1H 0AJ UK
[4]Peter Gore Department of Immunobiology, School of Immunology & Microbial Sciences, Kings College London, London SE1 9RT UK
[5]Division of Cell Biology, Science Faculty, Department of Biology, Utrecht University, 3584 CH Utrecht, The Netherlands
[6]Merus, LSI, Yalelaan 62, 3584 CM, Utrecht, The Netherlands
[7]Centre for Micro-Photonics, Faculty of Science, Engineering and Technology, Swinburne University of Technology, Hawthorn, Victoria 3122 Australia
[8]Department of Biochemistry and Molecular Biophysics, Columbia University, New York, NY 10032 USA
[9]Protein Phosphorylation Laboratory, The Francis Crick Institute, 1 Midland Road, London NW1 1AT, UK
[10]School of Cancer and Pharmaceutical Sciences, King's College London, New Hunt's House, Guy's Campus, London SE1 1UL, UK

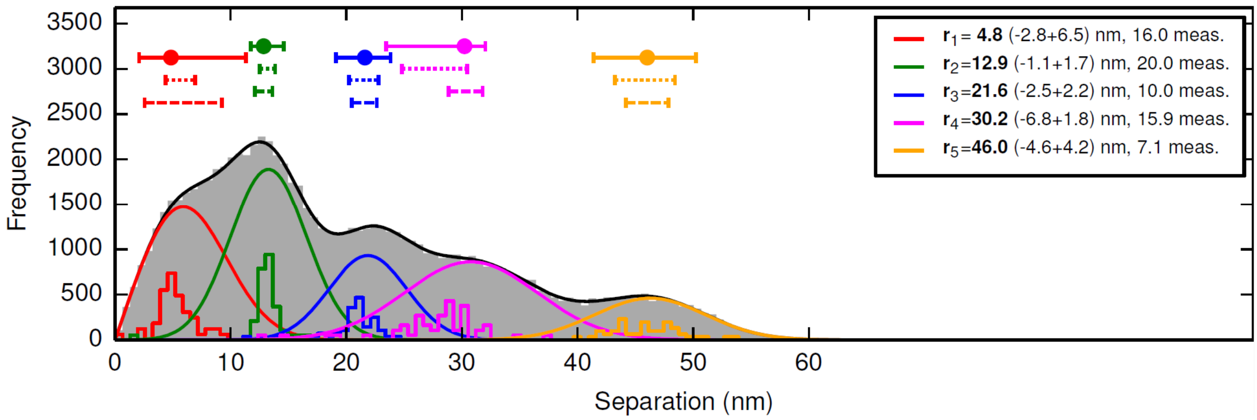

Supplementary Fig. 1 Statistics of the BIC and Bayesian parameter fit for ligand-free wtEGFR.
The figure shows the error estimate in the location of an individual isolated peak (dashed), the bootstrap-estimated error in the decomposition (dotted) and the combination of the two (solid). Means and errors together with the number of separations accumulated under each peak component are shown in the inset.

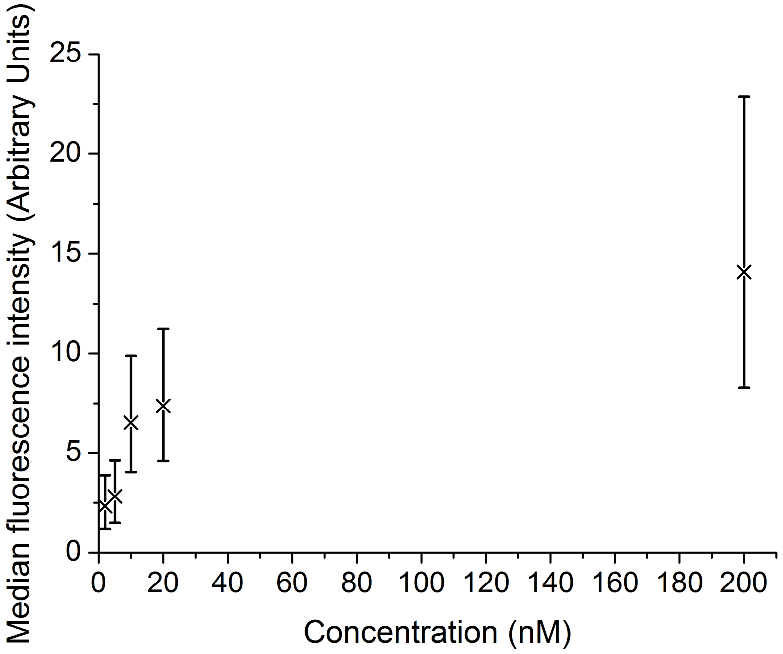

Supplementary Fig. 2 Affibody binding to EGFR on cells.

Median anti-EGFR Alexa 488-Affibody fluorescence in the membranes of intact CHO cells expressing wtEGFR at 4 x 10^5 receptors/cell measured by confocal microscopy, as a function of increasing Alexa 488-Affibody concentration. The error bars represent the standard deviation of the pixel values across the membranes of ~150 cells for a given Alexa 488-Affibody concentration. Data is taken from two biological replicates. The 4 nM Affibody concentration used labels ~20% of receptors.

a

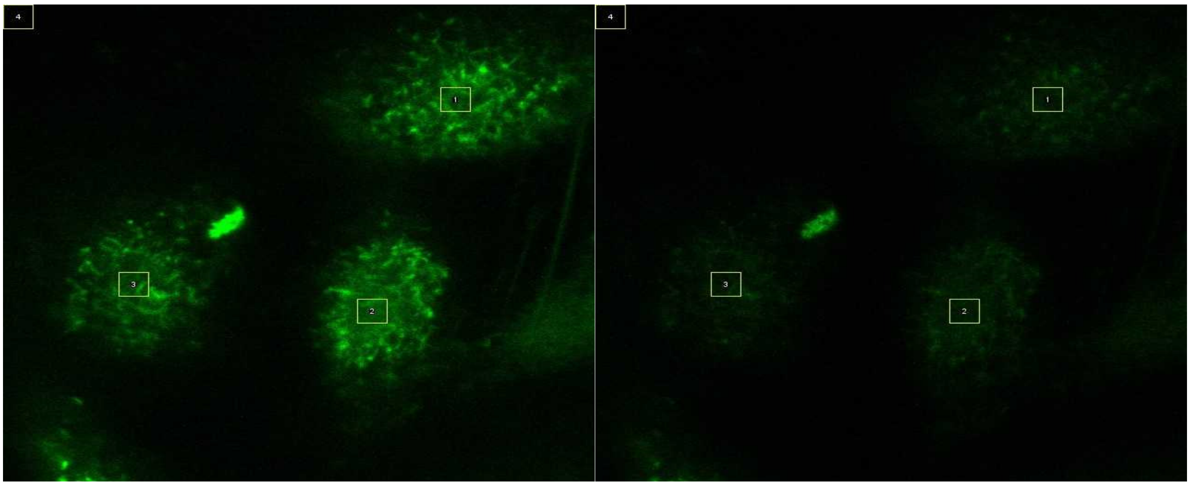

b

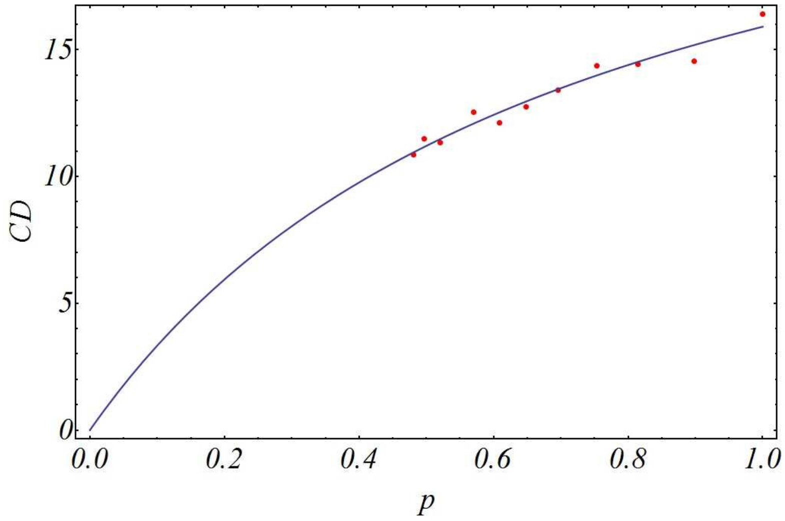

Supplementary Fig. 3 Example of pbICS data and analysis.

(a) Fluorescence confocal laser-scanning microscopy images of wtEGFR 100 nM Alexa 488-Affibody labelled CHO cells focussing on the apical membrane surface. Left image is before photobleaching and right image is after partial photobleaching. **(b)** pbICS data from region in (a). Vertical axis represents apparent cluster density and horizontal axis represents fractional fluorescence remaining. Solid line represents a model-free fit to the data using a monomer, dimer, trimer, tetramer, pentamer, hexamer, heptamer, octamer model. Parameters extracted from fit were: {c1,c2,c3,c4,c5,c6,c7,c8}:{0.518,0.291,0.190,<0.001,<0.001,<0.001,<0.001,<0.001}, where C1 is the normalised density of monomers, c2 is the normalised density of dimers etc. pbICS curve was obtained from ROI 2.

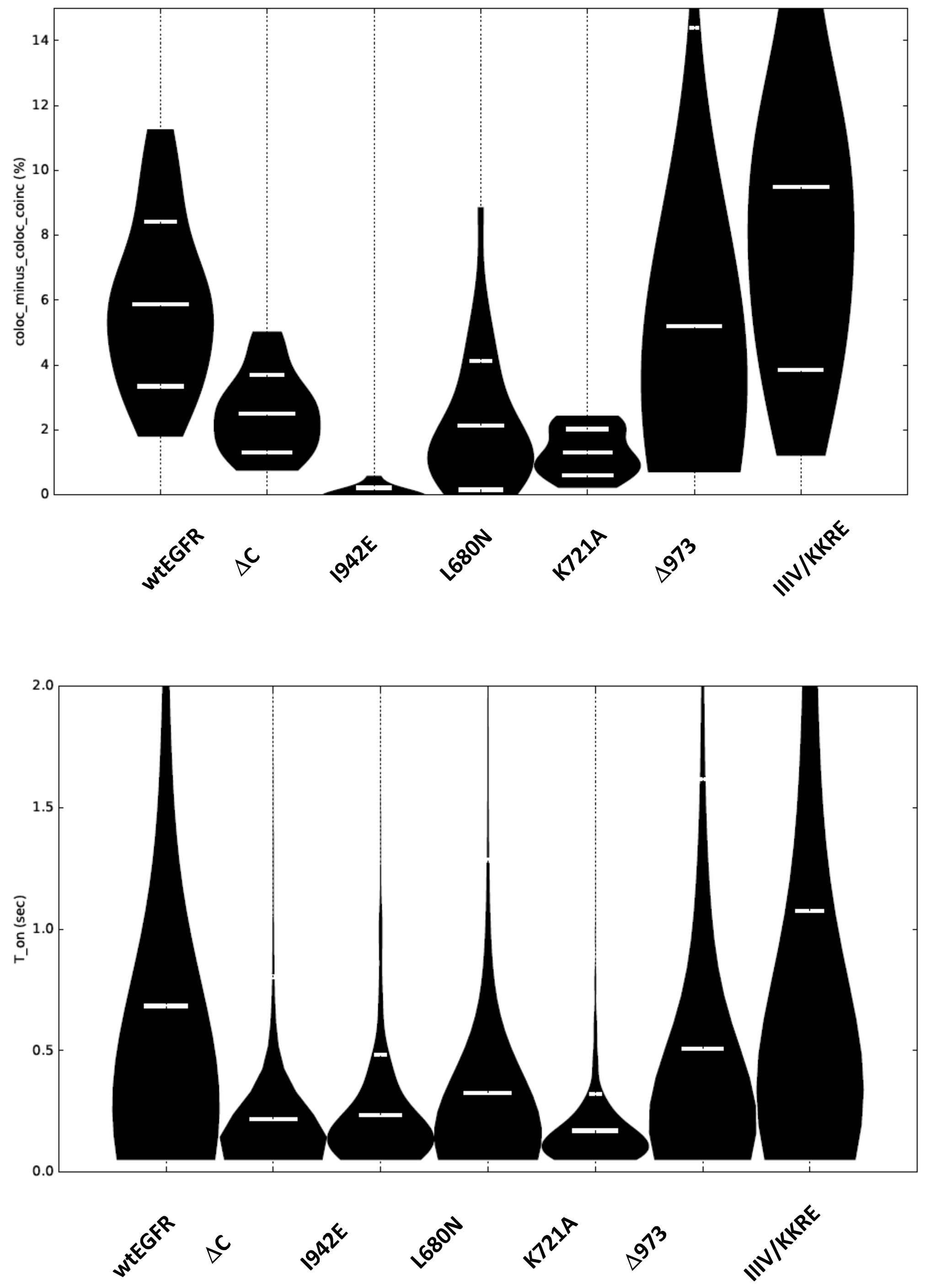

Supplementary Fig. 4 Single particle tracking screen of EGF-bound EGFR mutants.

Top panel: Fraction of tracks where two different-colour particles labelled with a mixture of CF640R-EGF/ Alexa 488-EGF spend ≥5 frames (250 ms) together (colocalised) within <1 pixel (denoted fraction of pairwise particle colocalisation events). Bottom panel: distribution of the duration of these pairwise interactions (τ_{ON}). Horizontal spreads separate data points (~5,000) within each condition. Horizontal white lines mark the mean and SD. Coincidental colocalisation statistics were accounted for[1]. The results show that ligand-bound I942E-EGFR forms much fewer *de novo* complexes.

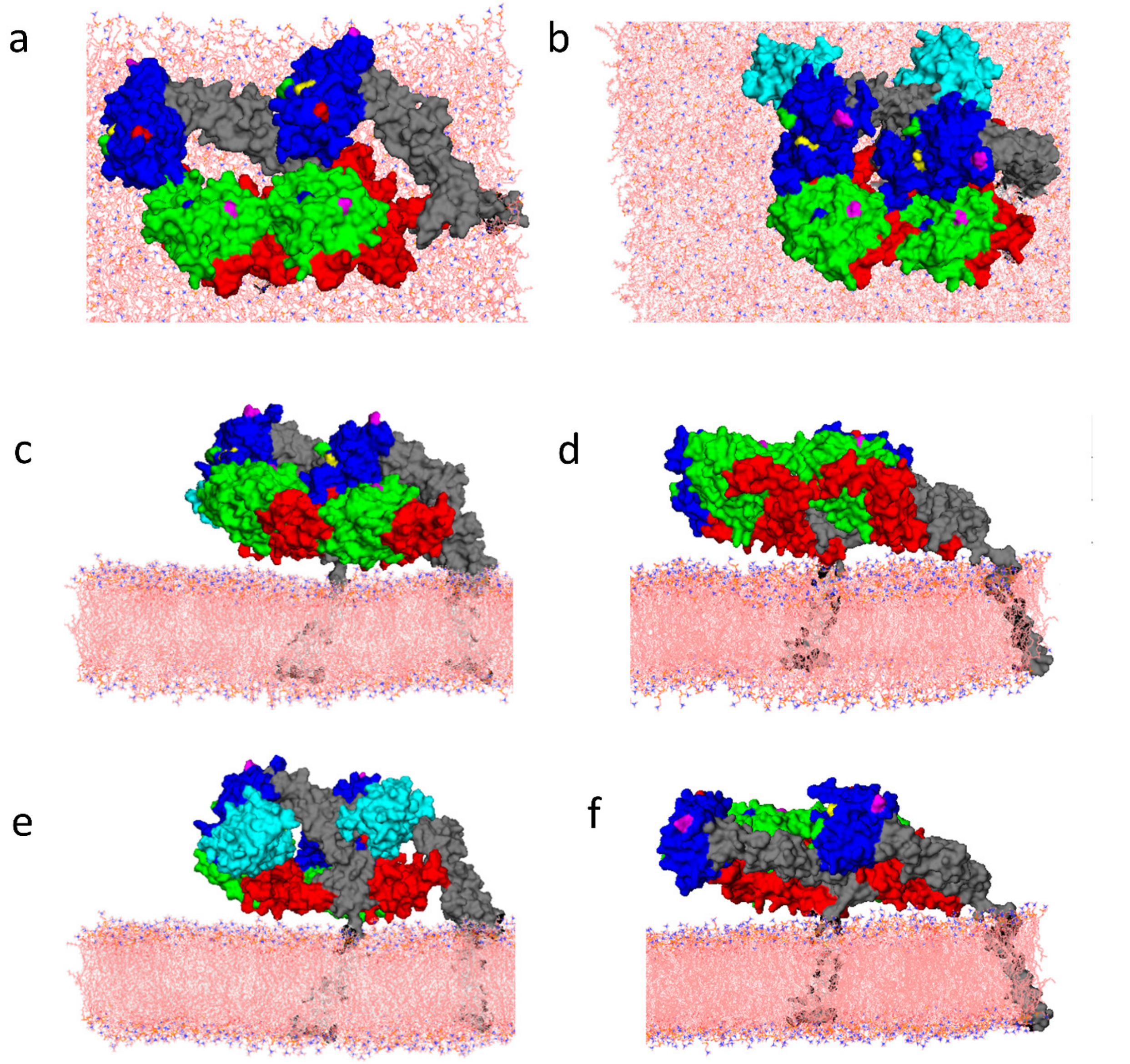

Supplementary Fig. 5 Lateral and vertical distances in the head-to-head dimer model.
Distance measurements between selected amino-acids of adjacent protomers in: **(a)** the naked head-to-head dimer without 9G8-NB bound and **(b)** the head-to-head dimer bound to 9G8-NB (cyan). Selected amino acids from which the measurements in Supplementary Tables 1-2 were derived are highlighted (red, yellow, green, magenta or blue). **(c-f)** Views of the extracellular domain highlighting selected amino acids from which vertical separations to the plasma membrane were measured (see Supplementary Table 3). In all panels: DI is represented in green, DII in red, DIII in blue and DIV in grey, while the membrane is depicted in pink.

residue	DIII (blue) distances (nm)	
	+9G8	-9G8
P^{362} (red)	4.27	6.67
Q^{384} (yellow)	4.24	6.94
H^{409} (green)	5.04	6.93
N^{473} (magenta)	5.90	8.03

Supplementary Table 1
Summary of the intra-dimer distances measured between the amino acids highlighted in Supplementary Fig. 5a.

residue	DI (green) distances (nm)	
	+9G8	-9G8
S^{99} (blue)	5.09	4.37
S^{153} (magenta)	4.74	4.14

Supplementary Table 2
Summary of the intra-dimer distances measured between the amino acids highlighted in Supplementary Fig. 5b.

Domain	Distances (nm)	
	-9G8	+9G8
I (E^{35})	3.93	1.87
I (N^{128})	6.48	4.44
III (F^{357})	5.73	2.99
III (K^{443})	5.46	6.25

Supplementary Table 3
Summary of the distances measured between the amino acids highlighted in Supplementary Fig. 5c-f and the plasma membrane.

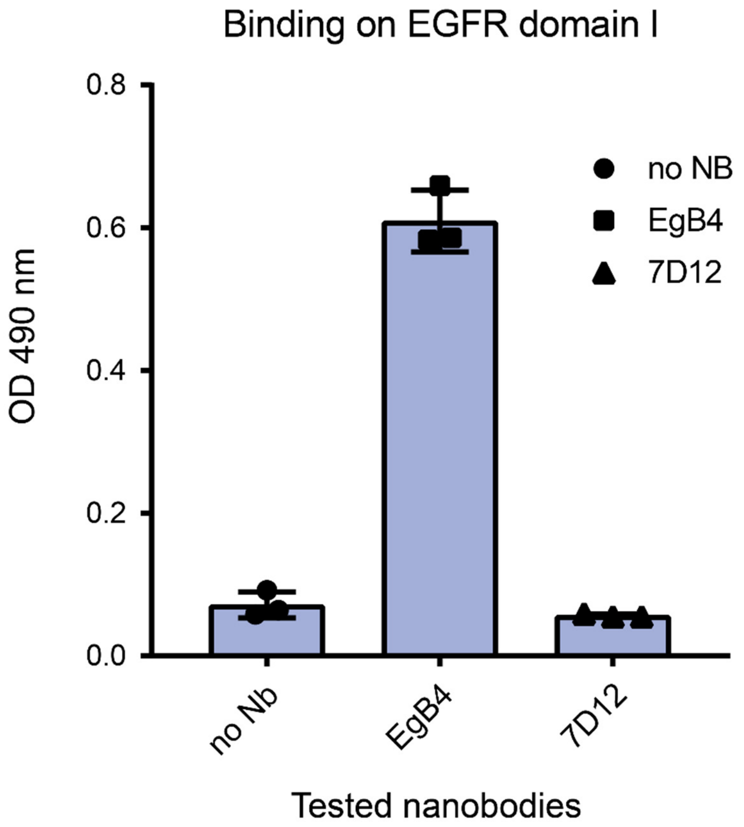

Supplementary Fig. 6 EgB4 Nanobody (NB) specificity for domain I of the ectodomain of wtEGFR. Differential binding of the indicated NBs to biotinylated DI of the wtEGFR ectodomain analysed by ELISA. The 7D12 anti-EGFR NB specific for DIII is used as control (Schmitz et al. 2013)[2]. The EgB4-NB binds EGFR without having an effect on EGF binding[3].

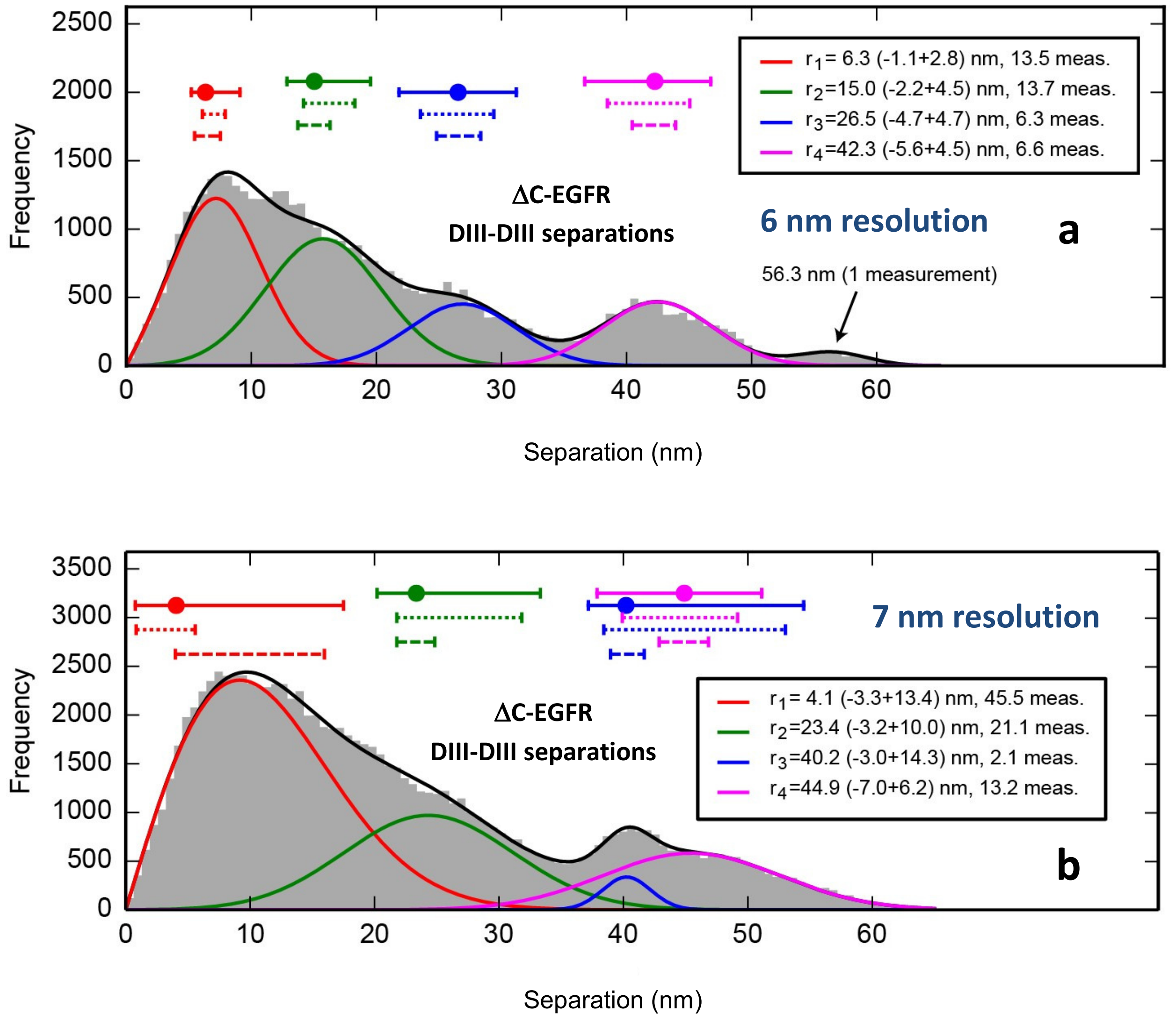

Supplementary Fig. 7 Statistics of the BIC and Bayesian parameter fit for ligand-free ΔC-EGFR.
(a) and **(b)**: FLImP distributions of DIII-DIII separations from ΔC-EGFR showing the error estimate in the location of individual isolated peaks (dashed), the bootstrap-estimated error in the decomposition (dotted) and the combination of the two (solid). Means and errors together with the number of separations accumulated under each peak component are shown in the inset. The number of separations, although an integer at source, is returned by the calculations as a fraction because of errors. FLImP is stochastic and can therefore report stoichiometric separations and ad hoc separations, the latter from receptors randomly near each other. In this context, note in (**a**) that the data retained at 6 nm resolution displays one FLImP separation measurement at 56.3 nm, which is larger than the longest predicted by the curved polymer chain model in Fig. 4a (main text). Given that in (**a**) the 4th component of the FLImP distribution (42 nm) is separated from the 56 nm position by 14 nm, we reasoned that, when ~2-fold more data are retained at 7 nm resolution **(b)**, if the isolated 56 nm separation detected was stoichiometric and significant, we should still resolve peak components at ~42 nm and ~56 nm. The absence of any evidence for a peak component longer than the ~45 nm predicted by the model at 7 nm resolution in (**b**) suggests that the isolated 56.3 nm separation observed at 6 nm resolution is a random receptor-receptor colocalisation event. Note that all the separations resolved at 7 nm are predicted by the head-to-head polymer model. The lower resolution of the 7 nm distribution in (**b**) means that not all short separations (separated by 7 nm) can be resolved.

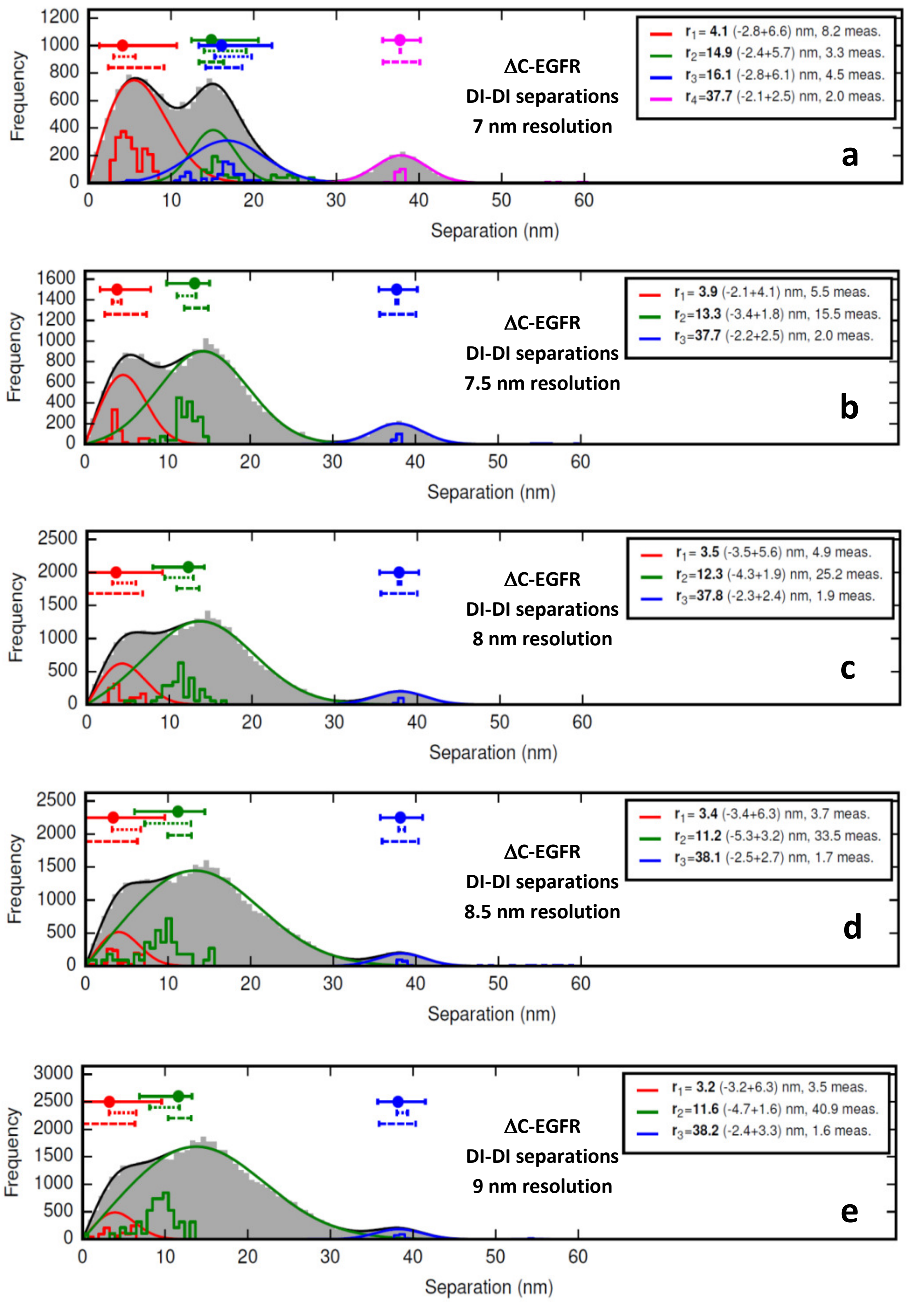

Supplementary Fig. 8 Statistics of the BIC and Bayesian parameter fit of DI-DI separations for ligand-free ΔC-EGFR.

(**a-e**): FLImP distributions of DI-DI separations from ΔC-EGFR showing the error estimate in the location of individual isolated peaks (dashed), the bootstrap-estimated error in the decomposition (dotted) and the combination of the two (solid). Means and errors together with the number of separations accumulated under each peak component are shown in the inset. The number of separations, although an integer at source, becomes a fraction because of errors in the calculations. Note in (**a**) that data retained at 7 nm resolution displays two DI-DI FLImP separation measurements consistent with 38 nm, which is longer than that predicted by the curved polymer chain model (31 nm) (Fig. 4a). Given that 38 nm is separated from the adjacent 16 nm component by 22 nm, it should still be resolved by FLImP distributions with resolutions between 7 nm and 9 nm (**a-e**). If the 38 nm position was stoichiometric and significant, when more data are retained at 7-9 nm resolutions, we would expect the 38 nm component to accumulate additional separations. Contrary to this, the 38 nm component becomes less significant as more data are accumulated. This is consistent with the two separations detected at 38 nm corresponding to random colocalisation events.

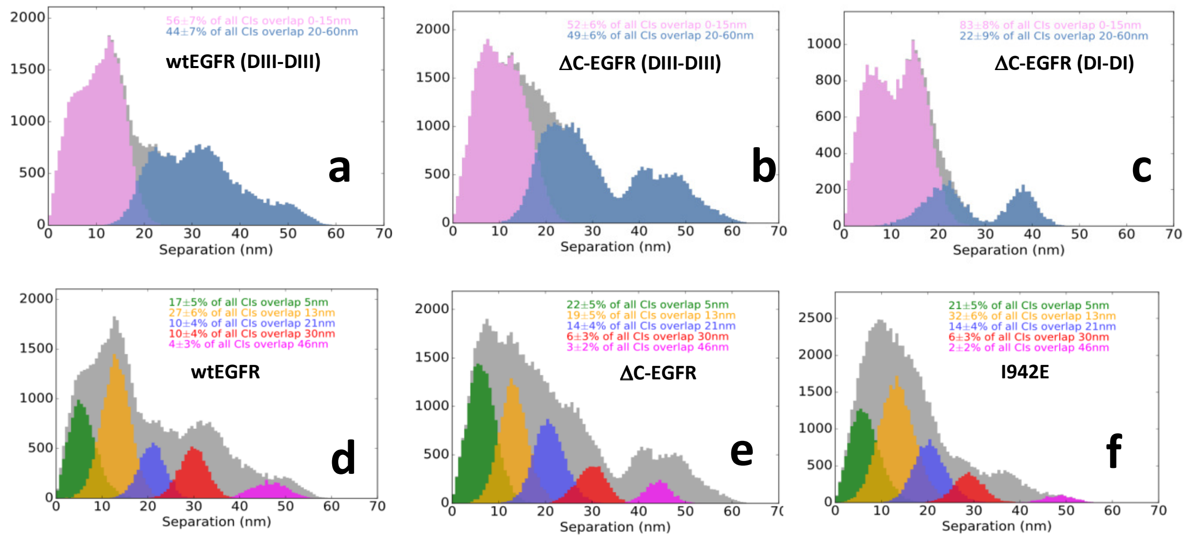

Supplementary Fig. 9 Fraction of separations consistent with a given hypothesis determined by FLImP experiments.

(a-c) Number of measurements consistent with the distances expected for dimers (<15 nm) and oligomers (20-60 nm). Errors were assesed with bootstrap-resampling[1]. The ratio of the number of measurements whose 69% CI overlap the range (0-15 nm) (pink) or (20-60 nm) (blue) to the total number of all FLImP measurements is shown. Note that the number of DI-DI separations longer than 20 nm are significantly fewer than DIII-DIII. **(d-f)** The distributions (green, yellow, blue, red, magenta) compiled from the FLImP measurements whose 69% CIs contain the DIII-DIII separations 5 nm, 13 nm, 21 nm, 30 nm or 46 nm. The ratio of the number of measurements whose 69% CI contain that distance to the total number of all FLImP measurements is shown. Error bars were calculated by bootstrap-resampling the data 1000 times with replacement and repeating the analysis[1]

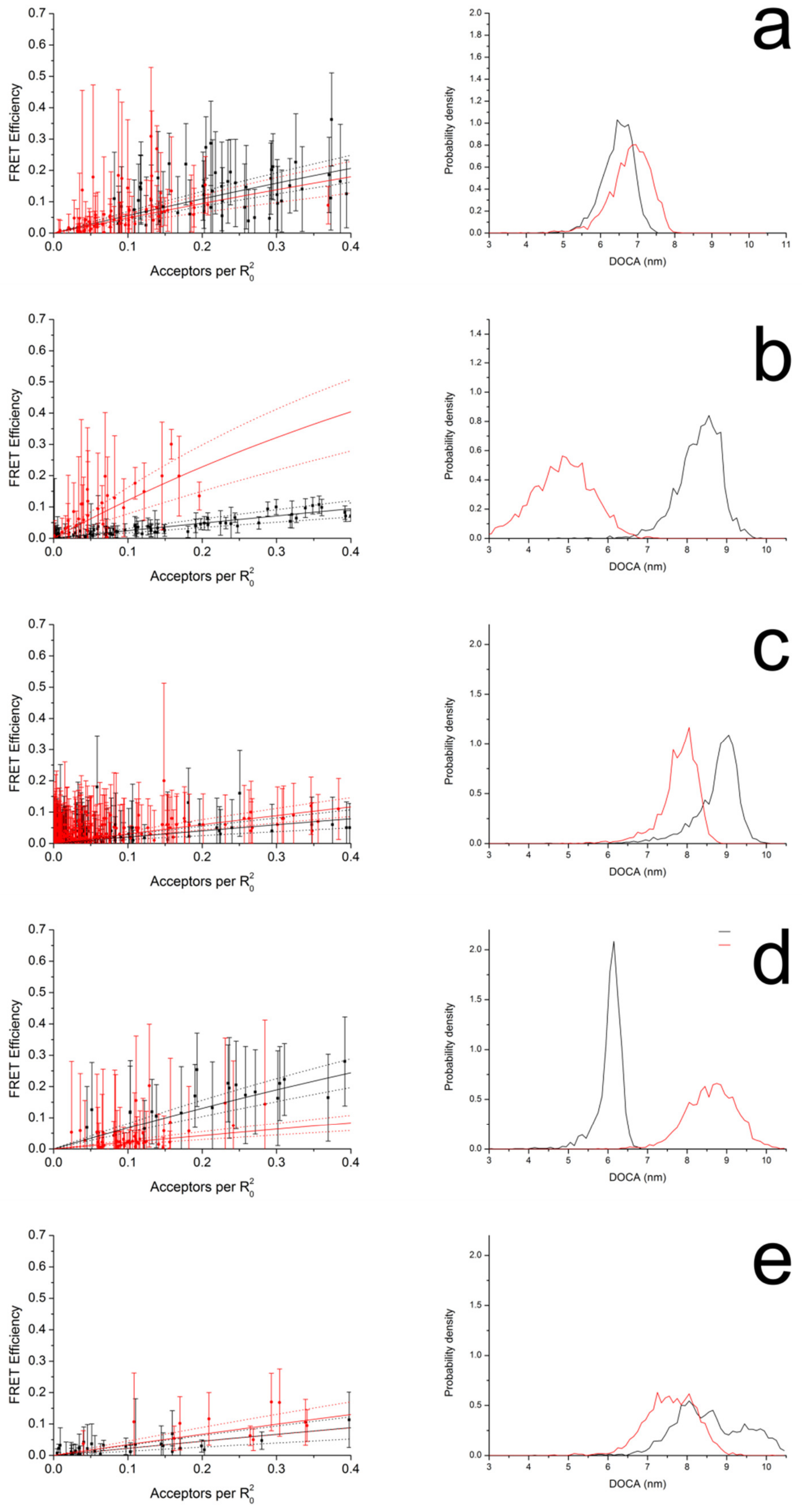

Supplementary Fig. 10 Distance of closest approach (DOCA) from DI and DIII to the cell surface.
FRET efficiency as a function of increasing membrane probe acceptor density for donor labelled **(a)** CHO cells expressing wtEGFR, **(b)** CHO cells expressing wtEGFR treated with 9G8-NB, **(c)** CHO cells expressing wtEGFR treated with erlotinib, **(d)** CHO cells expressing L680N-EGFR or **(e)** CHO cells expressing L834R-

EGFR. The donor was either Alexa 488-Affibody (DIII) (black) or Alexa 488-EgB4-NB (DI) (red). Each data point represents the mean acceptor density vs the mean FRET efficiency derived from the membrane pixels of a single cell. The error bars represent the standard deviation of the measured FRET efficiencies. The number of data points (each point from an individual cell) for wtEGFR + Affibody, wtEGFR + EgB4-NB, wtEGFR + 9G8-NB + Affibody, wtEGFR + 9G8-NB + EgB4-NB, wtEGFR + erlotinib + Affibody, wtEGFR + erlotinib + EgB4-NB, L680N-EGFR + Affibody, L680N-EGFR + EgB4-NB, L834R-EGFR + Affibody, and L834R-EGFR + EgB4-NB are: 54, 63, 97, 30, 119, 170, 40, 47, 30, and 11, respectively. Data is taken from at least two independent biological replicates. The distributions shown on the right of each panel represent the result of bootstrap regression analysis comparing the data to a model generated by simulating a donor above a plane of acceptors of increasing density with a Monte-Carlo simulation, as described in Tynan et al.[4]. Each bootstrap distribution was generated by random sampling of the original data with replacement before fitting to the FRET response model. For each condition 3000 bootstrap resamples were used. The model fit corresponding to the mean DOCA is plotted as a solid line. Dashed lines correspond to the upper and lower quartile of the corresponding bootstrap distributions.

	DI DOCA (nm)	DIII DOCA (nm)	test statistic	P-Value
wild type EGFR	6.73 ± 0.68	6.41 ± 0.65	0.32	0.2800
wild type EGFR + 9G8 NB	4.69 ± 0.89	8.22 ± 0.68	3.53	< 0.0010
wild type EGFR + erlotinib	7.72 ± 0.62	8.65 ± 0.94	0.93	0.0057
L680N-EGFR	8.50 ± 0.72	6.02 ± 0.45	2.48	< 0.0010
L834R-EGFR	7.48 ± 0.77	8.38 ± 1.03	0.88	0.1253

Supplementary Table 4 Numerical FRET results and error estimations

Mean bootstrap DOCA values and standard deviations for EGFR receptors labelled at DI with Alexa 488-EgB4-NB or DIII with Alexa 488-Affibody. P values represent the result of bootstrap hypothesis testing comparing the observed difference between the mean DI and DIII DOCA values in each experiment (the test statistic) with the null hypothesis that DI DOCA – DIII DOCA = 0. The null hypothesis can be rejected to the 0.01 significance level for all except wtEGFR and L834R-EGFR.

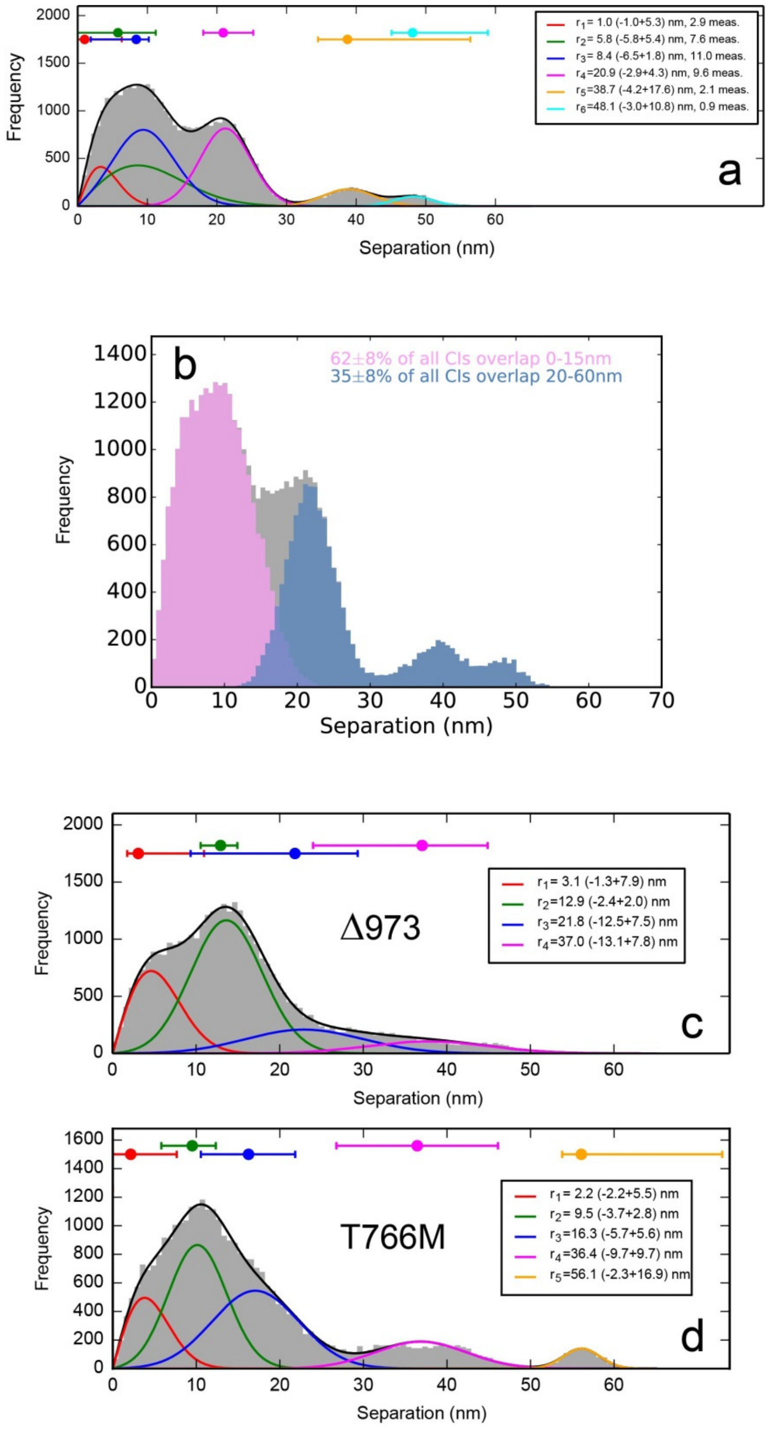

Supplementary Fig. 11 9G8-NB bound wtEGFR forms oligomers and analysis of Δ973 and T766M-EGFR. (a) FLImP distribution (grey) of DIII-DIII separations between CF640R-Affibody molecules bound to wtEGFR on CHO cells in the presence of 200 nM 9G8-NB compiled from 34 FLImP measurements with CI≤7nm, decomposed into a sum of six Rician components. The inset shows positions and error estimates[1]. **(b)** Number of measurements consistent with the distances expected for dimers (<15 nm) and oligomers (20-60 nm). Errors were assesed with bootstrap-resampling. The ratio of the number of measurements whose 69% CI overlap the range (0-15 nm) (pink) or (20-60 nm) (blue) to the total number of all FLImP measurements is shown. Error bars were calculated by bootstrap-resampling the data 1000 times with replacement and repeating the analysis[5]. **(c)** As (a) for Δ973-EGFR compiled from 29 FLImP measurements with CI≤7nm decomposed into a sum of four Rician components. **(d)** As (a) for T766M-EGFR compiled from 27 FLImP measurements with CI≤6.5nm decomposed into a sum of five Rician components.

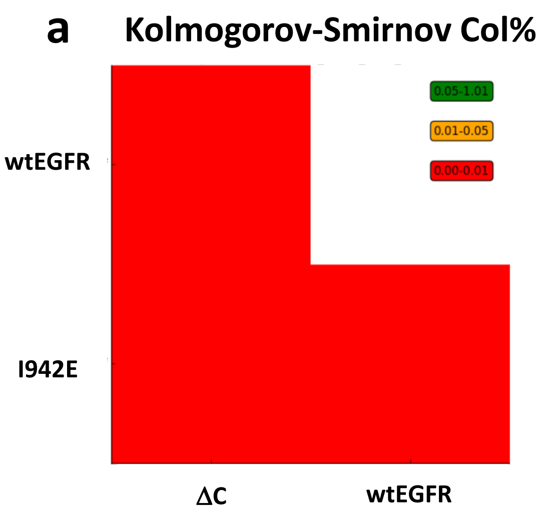

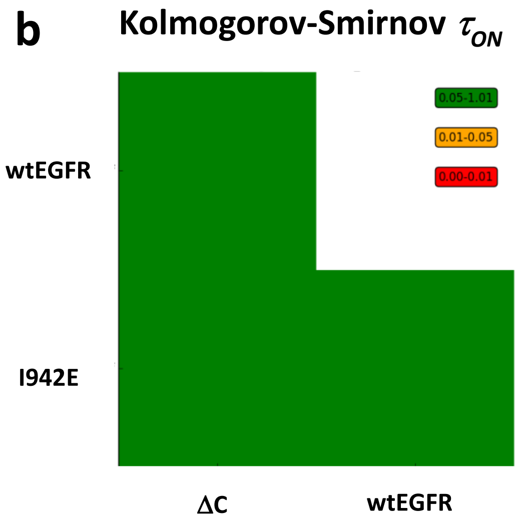

Supplementary Fig. 12 Significance of differences between compared conditions in two-colour SPT. The plots show tables from the Kolmogorov-Smirnov test P-value for the null hypothesis that particular pairs of measurement samples come from the same distribution. We cannot reject the hypothesis that pairs marked green are consistent with one another, while for pairs marked red we can say the measurements differ. **(a)** Results when comparing the colocalisation fractions displayed by the different receptors. **(b)** Results when comparing the duration (τ_{ON}) of the interactions.

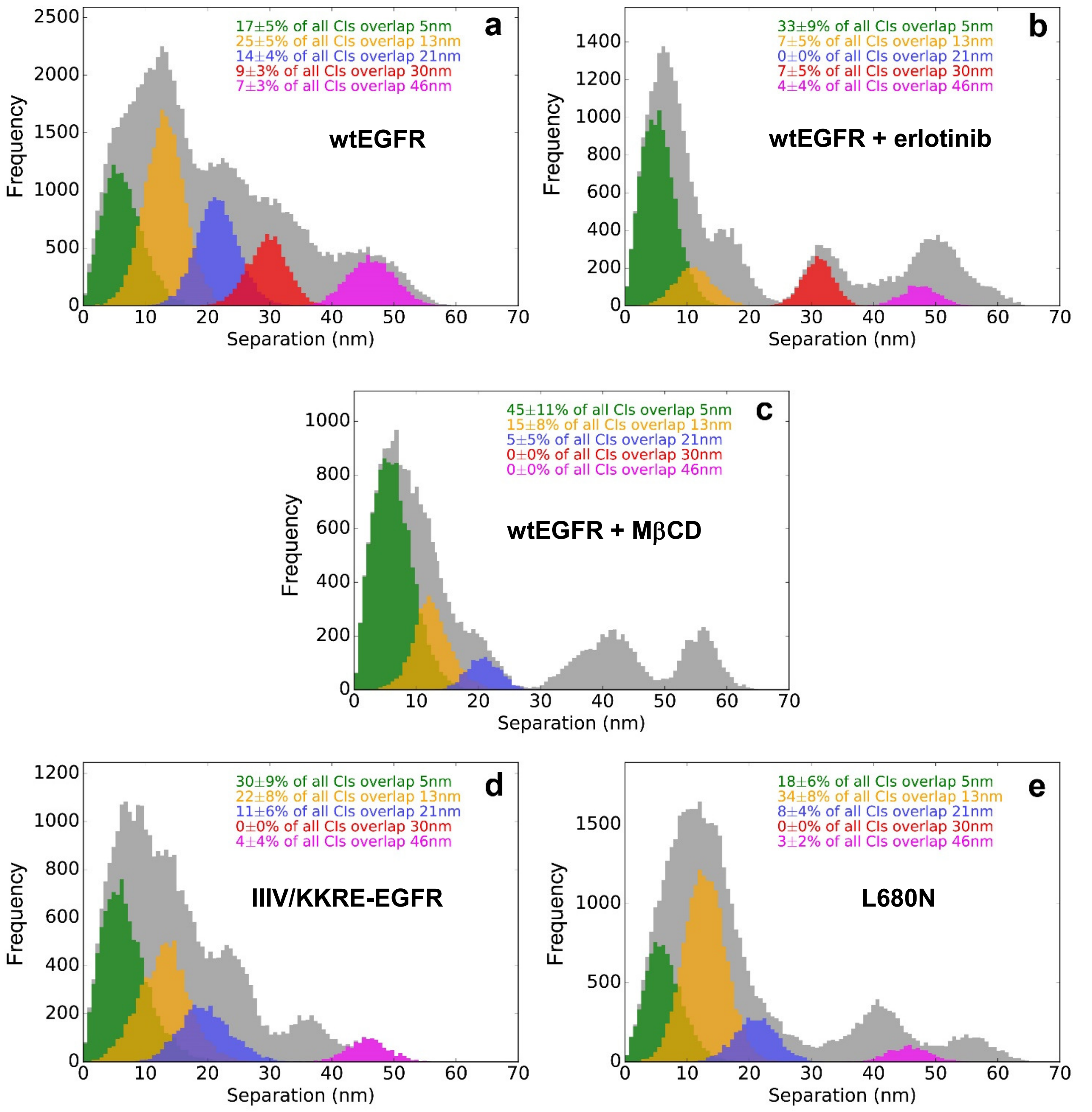

Supplementary Fig. 13 Fraction of separations consistent with the mean value of peak components. resolved in ligand-free wtEGFR.

(a) The FLImP distribution (grey) of ligand-free wtEGFR on CHO cells labelled with 4 nM CF640R-Affibody shown in Fig. 2b (main text) and the distributions (green, yellow, blue, red, magenta) compiled from the FLImP measurements whose 69% CIs contain the DIII-DIII separations 5 nm, 13 nm, 21 nm, 30 nm or 46 nm. The ratio of the number of measurements whose 69% CI contain that distance to the total number of all FLImP measurements is shown. Error bars were calculated by bootstrap-resampling the data 1000 times with replacement and repeating the analysis[1]. As (a) but on cells treated with **(b)** 1 μm erlotinib, **(c)** 10 mM MβCD. **(d)** The FLImP distribution (grey) of ligand-free IIIV/KKRE-EGFR mutant expressed on CHO cells. **(e)** As (d) but for the L680N-EGFR mutant.

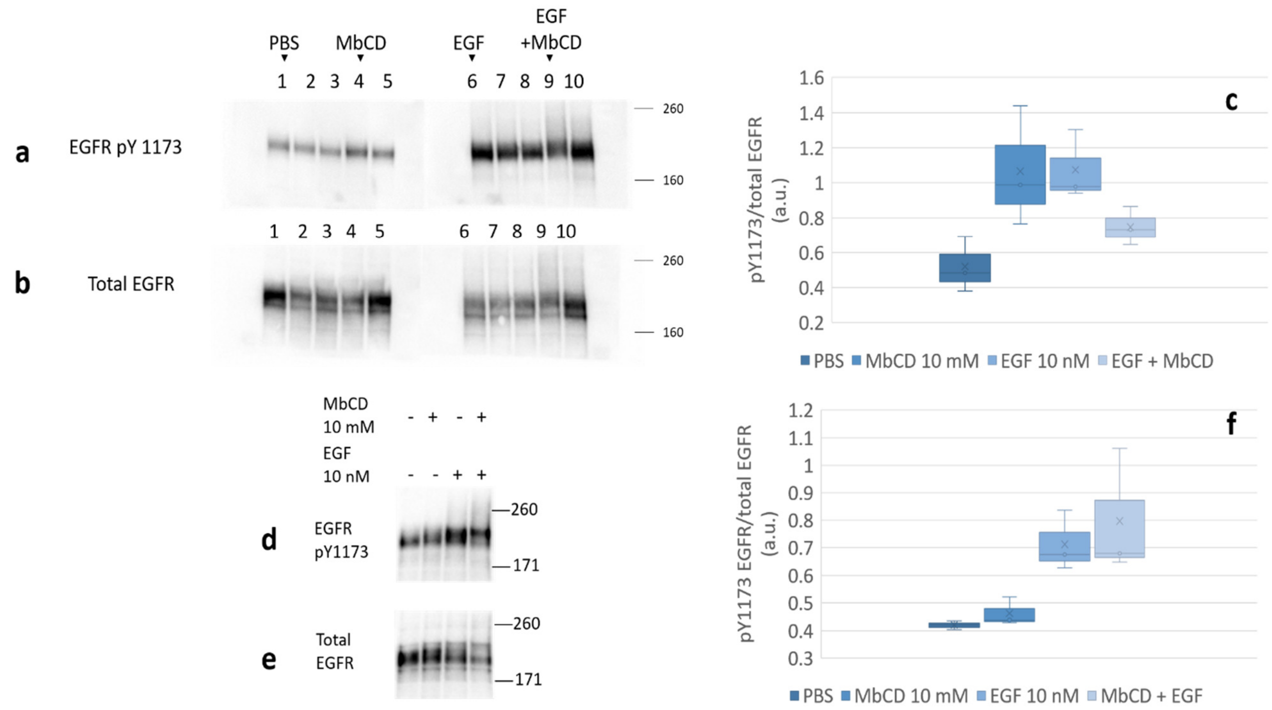

Supplementary Fig. 14 Phosphorylation of wtEGFR and IIIV/KKRE-EGFR in the presence and absence of cholesterol and EGF.

(a) Representative images of Western Blots used for the histogram in (c) showing CHO cells expressing wtEGFR treated with MβCD and other inhibitors (not discussed in this paper) in presence or absence of 10 nM EGF probed with anti-EGFR pY1173 antibody. **b)** Same blot re-probed after stripping with an anti-EGFR cocktail. **(c)** Box-and-whisker plot of C-terminal pTyr1173 auto-phosphorylation in CHO cells expressing wtEGFR calculated over n=3 replicates. The line represents the median (inclusive), the edges of the box the 25th and 75th quartile and the whiskers the fist and last quartile. **(d)** Representative images of Western Blots used for the histogram in (f) showing CHO cells expressing IIIV/KKRE-EGFR treated with MβCD in presence or absence of 10 nM EGF probed with anti-EGFR pY1173 antibody. **(e)** Same blot re-probed after stripping with an anti-EGFR cocktail. **(f)** Box-and-whisker plot of C-terminal pTyr1173 auto-phosphorylation in CHO cells expressing IIIV/KKRE-EGFR, as in panel (c), calculated over n=3 replicates.

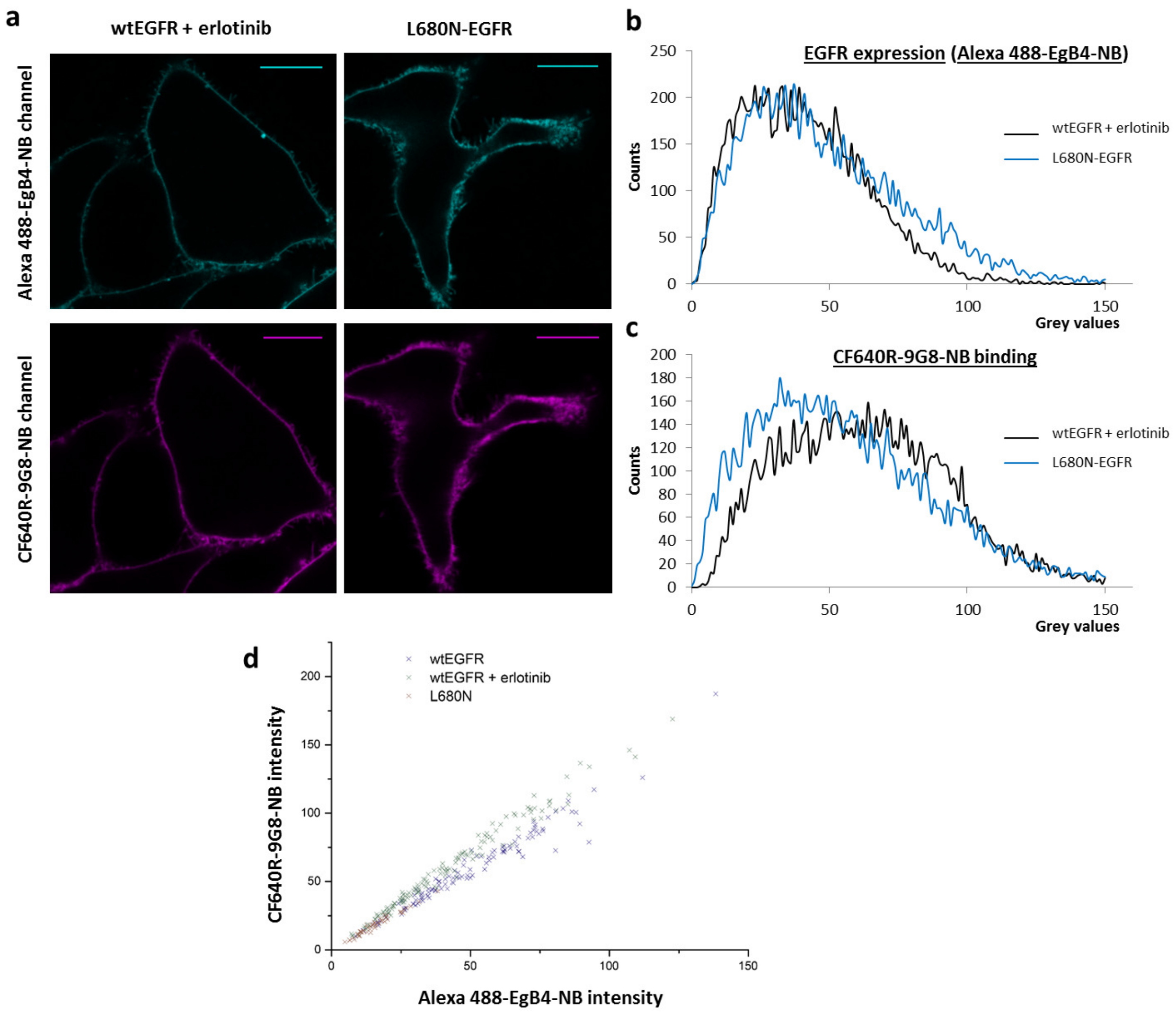

Supplementary Fig.15 Investigation of the tethered status of EGFR using 9G8-NB as a probe.

(a) Representative images from erlotinib treated wtEGFR expressing cells (left panel) and cells expressing L680N-EGFR (right panel). Top images show labelling with Alexa 488-EgB4-NB (cyan), which was used to probe the expression of the receptor, and bottom images show CF640R-9G8-NB binding (magenta). Scale bars = 10 µm. **(b- c)** Intensity histograms derived from the representative images in **(a)**. **(b)**, Intensity histograms of Alexa 488-EgB4-NB binding to wtEGFR + erlotinib (black) and L680N EGFR (blue) are shown. The corresponding histograms from CF640R-9G8-NB labelling are shown in **(c)**. **(d)** Scatter plot of the ratio between mean CF640R-9G8-NB and Alexa 488-EgB4-NB intensity of each cell, for all conditions investigated (wtEGFR, blue; wtEGFR + erlotinib, green; L680N-EGFR, red).

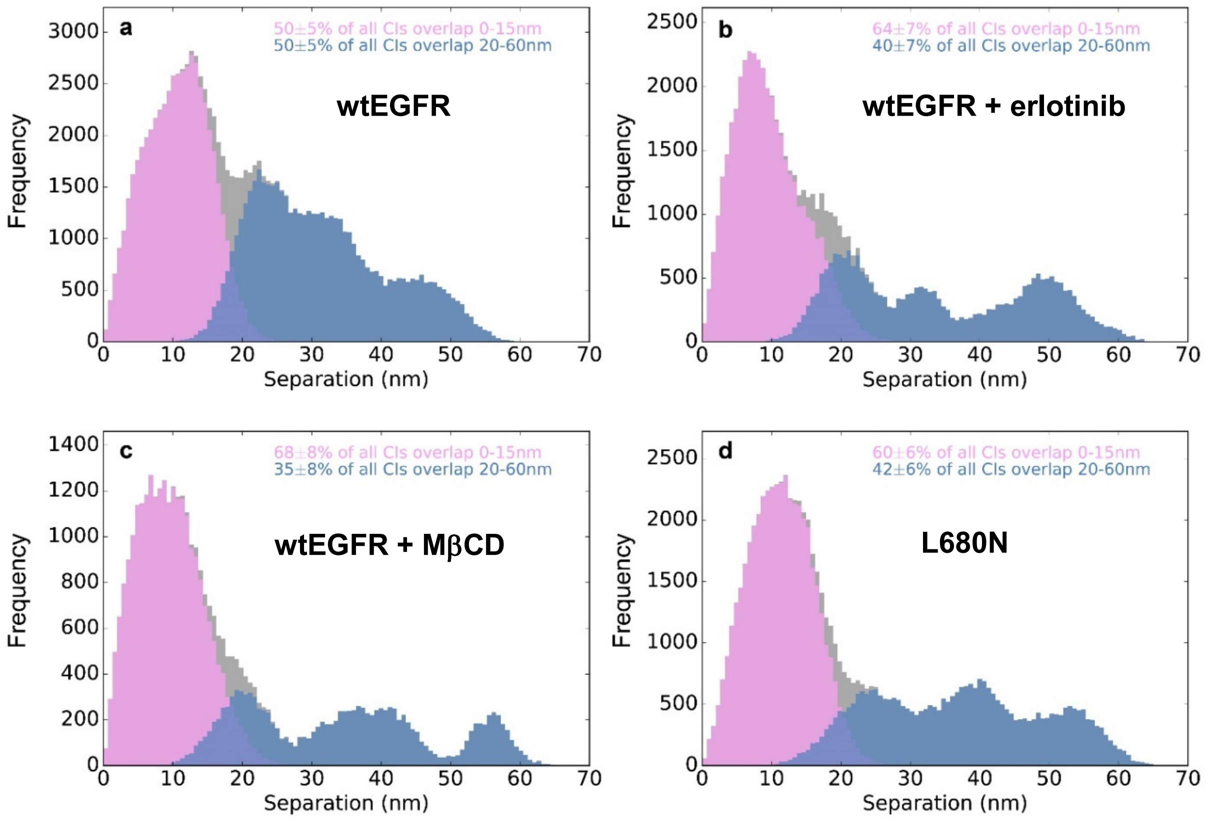

Supplementary Fig. 16 Low resolution (CI = 8 nm) FLImP histograms showing the conservation of the distribution profile and the fraction of separations consistent with dimers and oligomers.
(a) The FLImP distribution (grey) of ligand-free wtEGFR on CHO cells labelled with 4 nM CF640R-Affibody at lower (8 nm) resolution with ~1.5-2-fold more data. The ratio of the number of measurements whose 69% CI overlap the range 0-15 nm (pink) or 20-60 nm (blue) to the total number of all FLImP measurements is shown. Error bars were calculated by bootstrap-resampling the data 1000 times with replacement and repeating the analysis[1]. As (**a**) but on cells treated with **(b)** 1 μm erlotinib and **(c)** 10 mM MβCD. **(d)** On cells expressing the L680N-EGFR mutant. Results from the same conditions are shown in Fig. 2b, Fig. 5a, Fig. 5d, and Fig. 5l of the main text at 6-7 nm resolution.

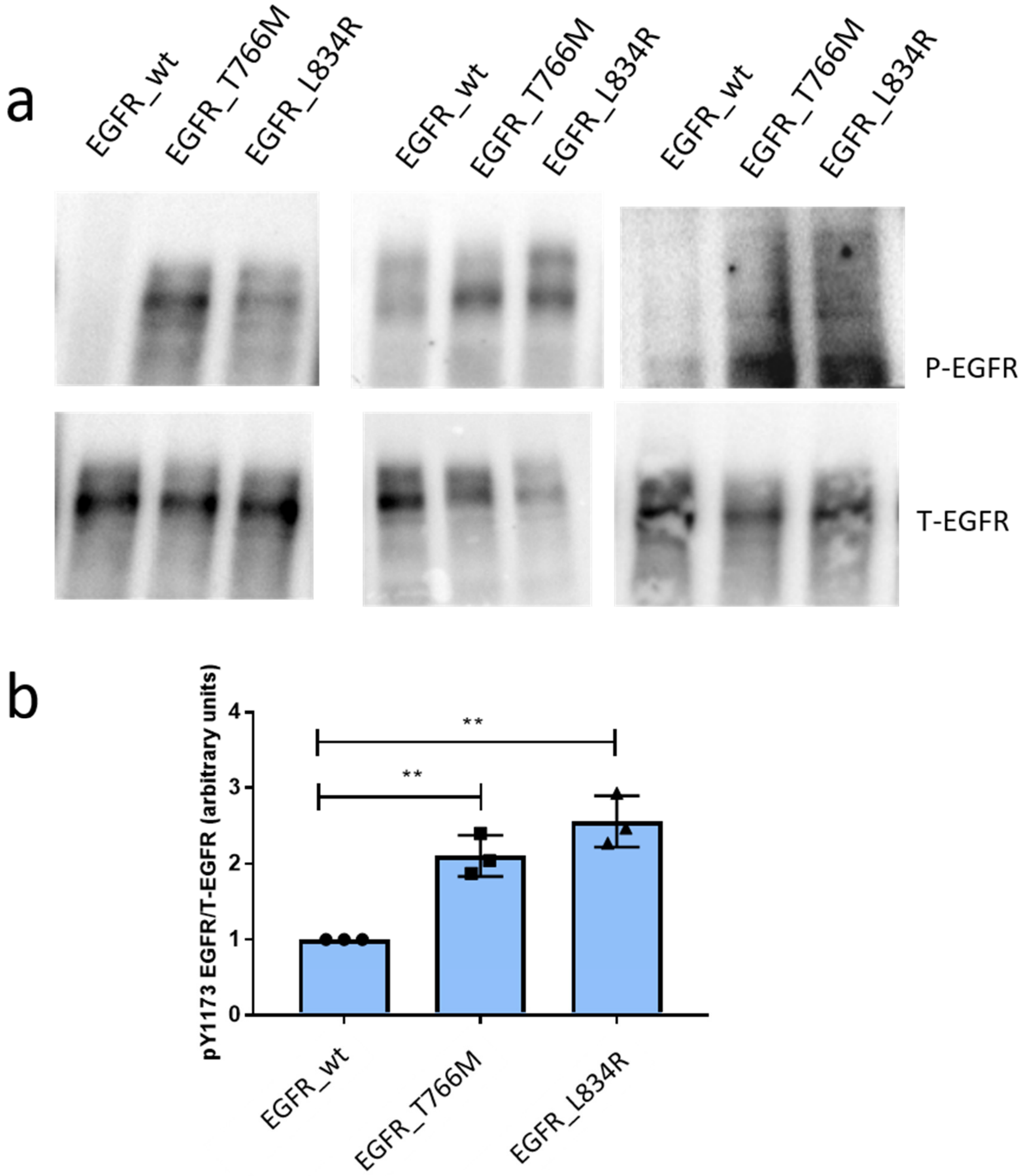

Supplementary Fig. 17 Basal phosphorylation of wtEGFR, T766M-EGFR and L834R-EGFR.
(a) Representative Western Blot images of CHO cells transfected with wtEGFR, T766M-EGFR or L834R-EGFR. Blots of pY1173 EGFR and total EGFR (T-EGFR) are shown in the figure. **(b)** Quantification of the Western blots shown in (**a**). Ratios were normalized using the value of wtEGFR as a baseline. Average ± SD calculated over n=3 replicates. Statistical analysis performed unpaired, two-tailed T-test: wtEGFR vs T766M-EGFR Pvalue = 0.0021, *t*= 7.062, df=4; wtEGFR vs L834R-EGFR Pvalue = 0.0013, *t*= 7.967, df=4.

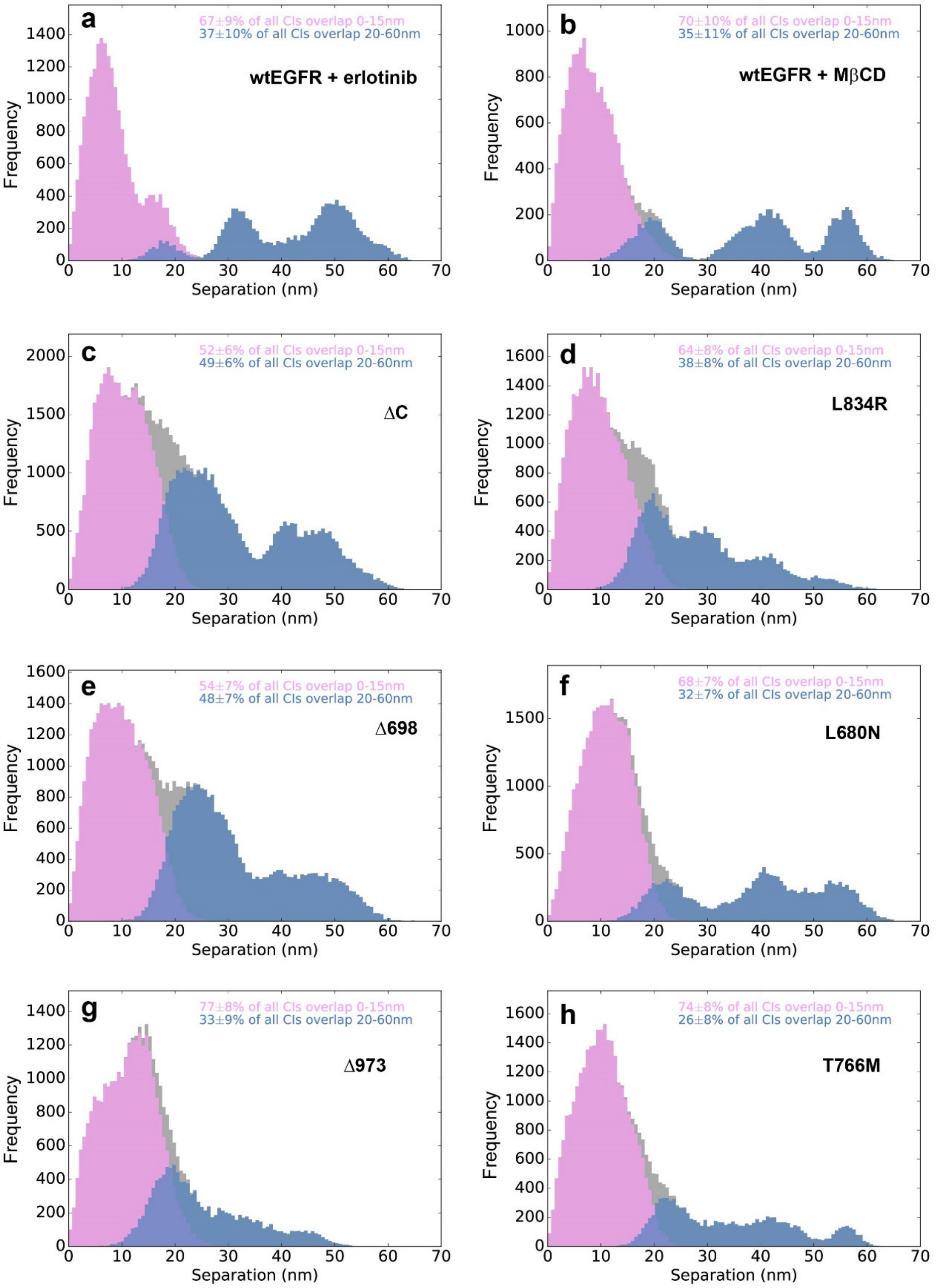

Supplementary Fig. 18 FLImP histograms showing the distribution profile and the fraction of separations consistent with dimers and oligomers.

Each condition **(a-h)** shows the FLImP distribution (grey) of ligand-free receptors labelled with 4 nM CF640R-Affibody at 6-7 nm resolution. The ratio of the number of measurements whose 69% CI overlap the range 0-15 nm (pink) or 20-60 nm (blue) to the total number of all FLImP measurements is shown. Error bars were calculated by bootstrap-resampling the data 1000 times with replacement and repeating the analysis[1]. The corresponding bar chart is shown in Fig. 6b, main text.

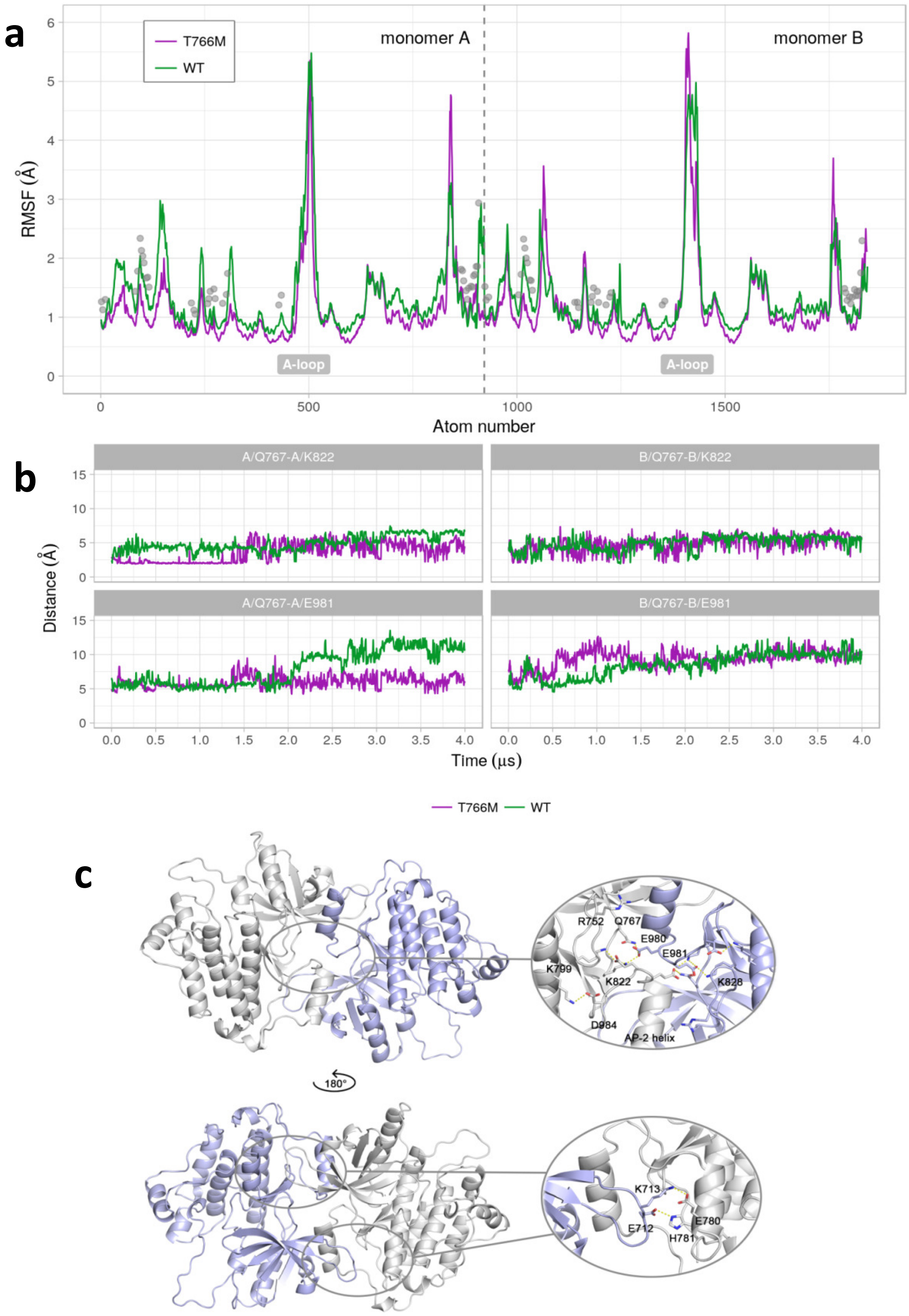

Supplementary Fig. 19 MD simulations of the T766M kinase domain.

(a) RMSF profiles for the WT (green) and T766M mutant sTKD (magenta). Interface residues are marked by grey circles. **(b)** Rolling average of selected distances at the dimer interface. The smoothing window is 5 ns. **(c)** The symmetric EGFR dimer shown from both sides to highlight the interface residues.

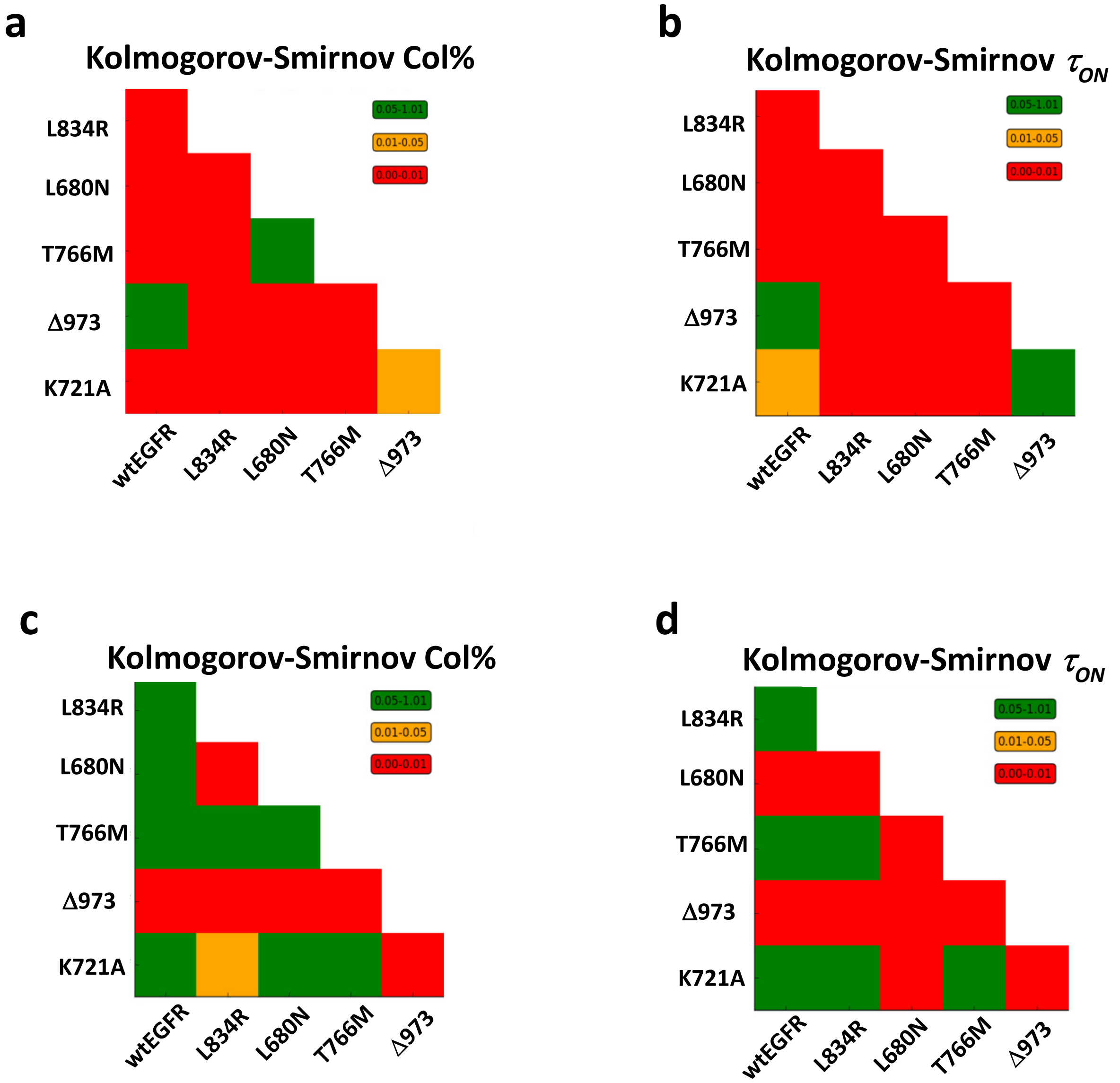

Supplementary Fig. 20 Significance of differences between compared conditions.
The plots show tables from the Kolmogorov-Smirnov test P-value for the null hypothesis that particular pairs of measurement samples come from the same distribution. We cannot reject the hypothesis that pairs marked green are consistent with one another, while for pairs marked red we can say the measurements differ. **(a)** and **(c)** Results when comparing the colocalisation fractions displayed by the different receptor mutants. **(b)** and **(d)** Results when comparing the duration (τ_{ON}) of the interactions in absence or presence of MβCD treatment, respectively.

Primer	Sequence
L843R SDM Fw	GTC AAG ATC ACA GAT TTT GGG cgg GCC AAA CTG CTG GGT GCG
L834R SDM Rev	CGC ACC CAG CAG TTT GGC ccg CCC AAA ATC TGT GAT CTT GAC
T766M SDM Fw	CC TCC ACC GTG CAG CTC ATC atg CAG CTC ATG CCC TTC GGC
T766M SDM Rev	GCC GAA GGG CAT GAG CTG cat GAT GAG CTG CAC GGT GGA GG
IIIV/KKRE sub-cloning Fw	agg aga tat acc atg CGA CCC TCC GGG ACG
IIIV/KKRE sub-cloning Rev	gtg atg gtg atg ttt TCA TGC TCC AAT AAA TTC ACT GC

Supplementary Table 5

Sequences of the primers used in the manuscript. Mutation sites are shown in lowercase and underlined for L834R-EGFR and T766M-EGFR SDM plasmids. Infusion tags are shown in lowercase and doubly underlined for IIIV/KKRE sub-cloning plasmids.

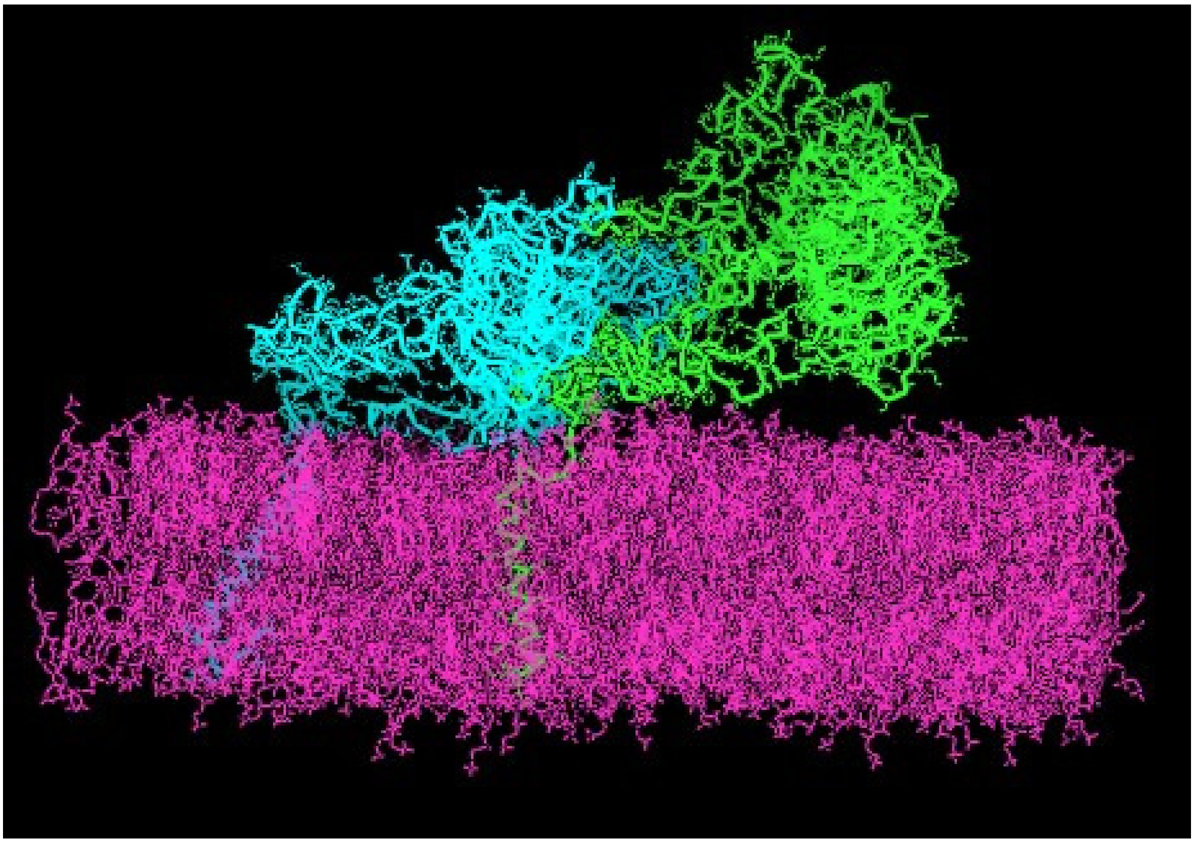

Supplementary Fig. 21 MD simulation after 13.8 µs, starting from the asymmetric dimer seen in the crystal packing of 4KRP, after removing 9G8-NB and adding the TM helix and the lipid bilayer.

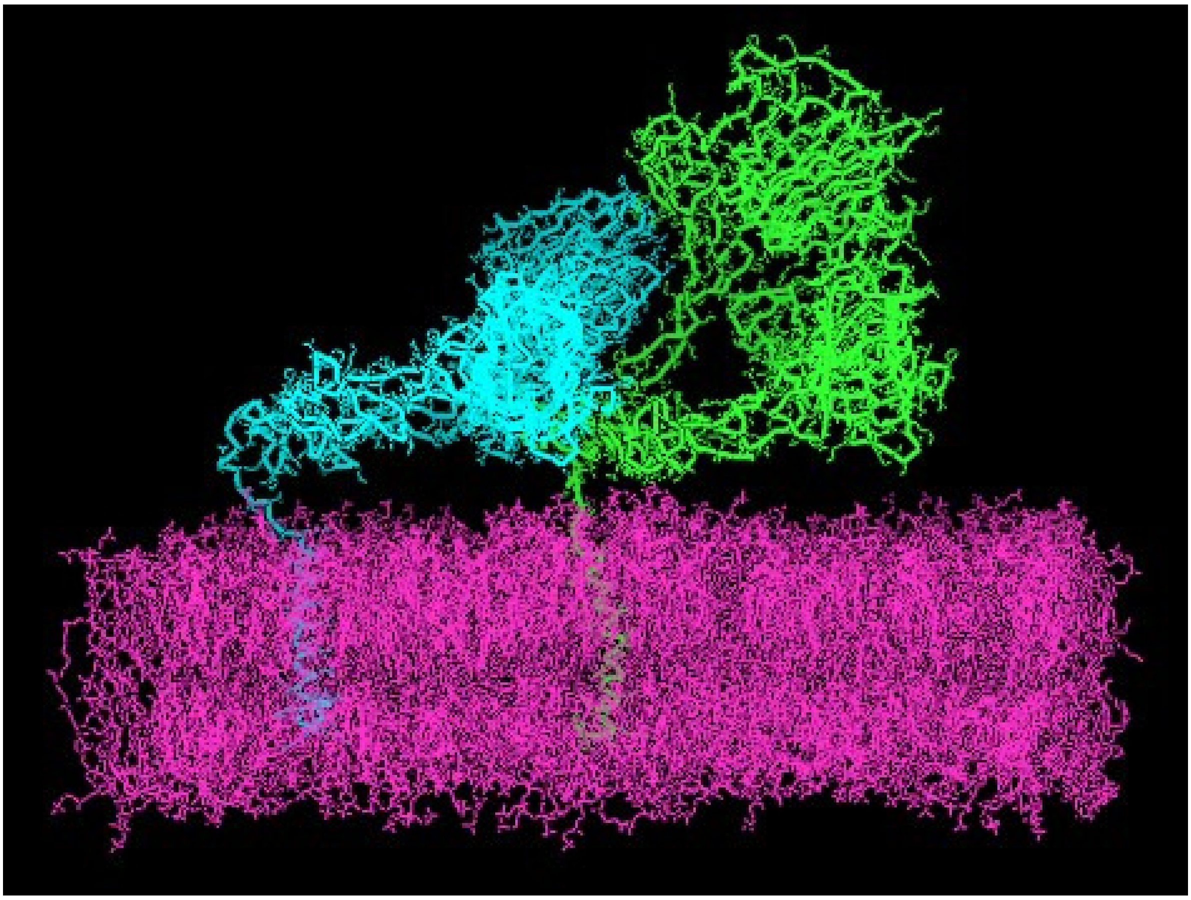

Supplementary Fig. 22 MD simulation after 20 µs, starting from the asymmetric dimer seen in the crystal packing of 4KRP, after removing 9G8-NB and adding the TM helix and the lipid bilayer.

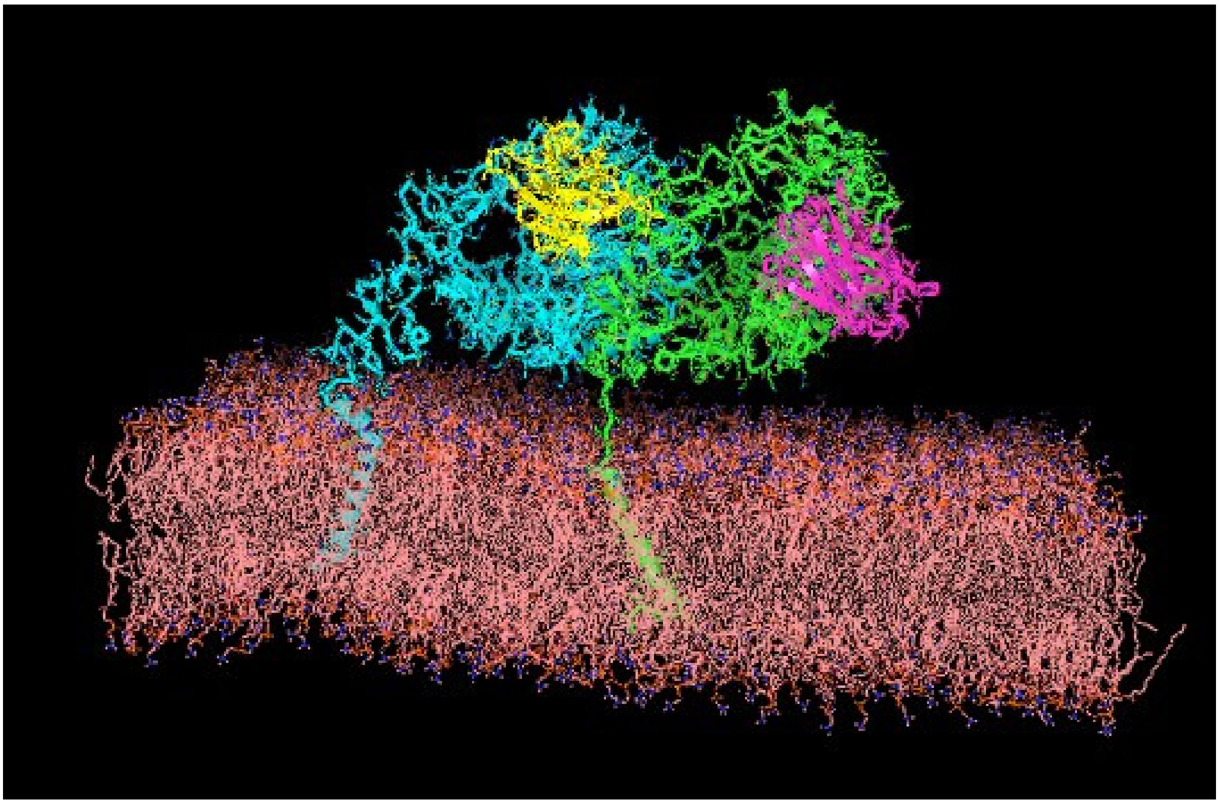

Supplementary Fig. 23 MD simulation after 20 μs, starting from the asymmetric dimer seen in the crystal packing of 4KRP, after adding the 9G8-NBs to the starting ECM dimer.

Supplementary Methods

Energy Calculations from the Simulations of the T766M Kinase Domain

For the unbiased MD simulations the energy of the simulated systems was initially minimized in 2 cycles of steepest-descent energy minimization. Each system was equilibrated using the following protocol: the initial velocities for the atoms were taken from Maxwell distribution at 310 K and the system was simulated for 10 ns at constant volume using velocity rescale thermostat[6] and position restraints on heavy atoms (1000 kJ mol^{-1} nm^{-2}), followed by 10 ns under the same conditions with position restraints only on Cα atoms (500 kJ mol^{-1} nm^{-2}). The system was equilibrated for additional 20 ns under constant pressure using Berendsen barostat[7], followed by 20 ns using Parrinello-Rahman barostat[8]. The production simulations were generated using GROMACS 5.1.4 biomolecular simulation package[9] with a 2-fs integration step, constant temperature of 310 K using velocity rescale thermostat1, and constant pressure of 1 bar using Parrinello-Rahman barostat[8]. Bond lengths were constrained using LINCS[10], while van der Waals interactions were treated with a cutoff of 10 Å. Electrostatic interactions were computed using the particle mesh Ewald method[11] with the direct sum cutoff of 10 Å and the Fourier spacing of 1.2 Å. Each production run was 4 μs long with the coordinate output every 100 ps. Trajectories were analysed using standard GROMACS tools. The analysis of the free-energy basins was performed as follows**:** all the structures that fell within basins selected for further analysis were clustered using the gromos algorithm[12] by using the RMSD of Cα atoms as the distance between the structures and the cutoff value of 2 Å. The central structure of the most populated cluster of each basin (i.e., the structure with the smallest distance to all the other members of the cluster) was chosen as the representative of the basin.

Supplementary References

1 Needham, S. R. et al. EGFR oligomerization organizes kinase-active dimers into competent signalling platforms. *Nat. Commun.* **7**, doi:10.1038/ncomms13307 (2016).

2 Schmitz, K. R., Bagchi, A., Roovers, R. C., van Bergen en Henegouwen, P. M. & Ferguson, K. M. Structural evaluation of EGFR inhibition mechanisms for nanobodies/VHH domains. *Structure* **21**, 1214-1224 (2013).

3 Hofman, E. G. et al. EGF induces coalescence of different lipid rafts. *J. Cell Sci.* **121**, 2519-2528 (2008).

4 Tynan, C. J. et al. Human epidermal growth factor receptor (EGFR) aligned on the plasma membrane adopts key features of drosophila EGFR asymmetry. *Mol. Cell Biol.* **31**, 2241-2252 (2011).

5 Bussi, G., Donadio, D. & Parrinello, M. Canonical sampling through velocity rescaling. *J. Chem. Phys.* **126**, doi:10.1063/1.2408420 (2007).

7 Berendsen, H. J. C., Postma, J. P. M., van Gunsteren, W. F., Di Nola, A. & Haak, J. R. Molecular-dynamics with coupling to an external bath. *J. Chem. Phys.* **81**, 3684-3690, doi:10.1063/1.448118 (1984).

8 Parrinello, M. & Rahman, A. Polymorphic transitions in singlecrystals: a new molecular dynamics method. *J. Appl. Phys.* **52**, 7182-7190, doi:10.1063/1.328693 (1981).

9 Abraham MJ, et al. GROMACS: High performance molecular simulations through multi-level parallelism from laptops to supercomputers. *SoftwareX*, **1–2**: 19-25 (2015).

10 Hess, B., Bekker, H., Berendsen, H. J. C. & Fraaije, J. G. E. M. LINCS: A linear constraint solver for molecular simulations. *J. Comp. Chem.* **18**, 1463-1472 (1997).

11 Essmann, U., Perera L & Berkowitz, M.L. . A smooth particle mesh ewald method. *J. Chem. Phys.* **103**, 8577-8593, doi:10.1063/1.470117 (1995).

12 Daura, X. et al. Peptide folding: When simulation meets experiment. *Angew. Chem. In. Ed.* **38**, 236-240 (1999).
