# Supplementary material for: The architecture of EGFR’s basal complexes reveals autoinhibition mechanisms in dimers and oligomers
Source: Nat Commun. 2018 Oct 18;9:4325. doi: 10.1038/s41467-018-06632-0 (PMC6193980; doi:10.1038/s41467-018-06632-0)
Supplement: Supplementary file 3 — Description of Additional Supplementary Files [file 41467_2018_6632_MOESM3_ESM.pdf]

Description of Additional Supplementary Files

File Name: Supplementary Data 1
Description: MD simulation after 13.8 µs, starting from the asymmetric dimer seen in the crystal packing of 4KRP, after removing 9G8-NB and adding the TM helix and the lipid bilayer.

File Name: Supplementary Data 2
Description: MD simulation after 20 µs, starting from the asymmetric dimer seen in the crystal packing of 4KRP, after removing 9G8-NB and adding the TM helix and the lipid bilayer.

File Name: Supplementary Data 3
Description: MD simulation after 20 µs, starting from the asymmetric dimer seen in the crystal packing of 4KRP, after adding the 9G8-NBs to the starting ECM dimer.
